# Housing insecurity pathways to physiological and epigenetic manifestations of health among aging adults: a conceptual model

**DOI:** 10.3389/fpubh.2025.1485371

**Published:** 2025-01-23

**Authors:** Aarti C. Bhat, Andrew Fenelon, David M. Almeida

**Affiliations:** ^1^Department of Human Development and Family Studies, The Pennsylvania State University, University Park, PA, United States; ^2^Center for Healthy Aging, The Pennsylvania State University, University Park, PA, United States; ^3^Population Research Institute, The Pennsylvania State University, University Park, PA, United States; ^4^School of Public Health, University of Minnesota-Twin Cities, Minneapolis, MN, United States; ^5^Minnesota Population Center, University of Minnesota-Twin Cities, Minneapolis, MN, United States; ^6^Life Course Center, University of Minnesota-Twin Cities, Minneapolis, MN, United States

**Keywords:** housing insecurity, stress process, aging, chronic conditions, allostatic load, epigenetics

## Abstract

**Introduction:**

Housing insecurity is a social determinant of health, as evidenced by its associations with mental, physical, and biological outcomes. The scientific understanding of the mechanisms by which housing insecurity is associated with health is still limited. This review adapts existing stress process models to propose a conceptual model illustrating potential pathways linking the specific stressor of housing insecurity to physiological and epigenetic manifestations of stress among aging adults.

**Methods:**

This narrative review examines literature across multiple fields, including public health, psychology, and sociology. The literature selected for this review was identified through scientific databases including Web of Science, PubMed, JSTOR, and Google Scholar; primarily peer-reviewed empirical studies, literature reviews, and research reports published in English between 1981 and 2024; and principally based in the United States context. A synthesis of this literature is presented in a proposed conceptual model.

**Results:**

The literature supports the existence of two main predictors of housing insecurity: sociodemographic characteristics and the historical/current context. The main mediating pathways between housing insecurity and manifestations of stress include health behaviors, psychosocial resources, and structural resources. Moderating factors affecting the associations between housing insecurity and manifestations of stress include government assistance, chronic discrimination/unfair treatment, and individual differences. These interdependent mediating and moderating mechanisms affect stressor reactivity, a proximal manifestation of stress, which contributes to the physiological and epigenetic distal manifestations of stress in aging adults.

**Discussion and implications:**

The prevalence of housing insecurity among aging adults is growing in the United States, with significant implications for public health and health disparities, given the growing percentage of aging adults in the population. Further empirical testing of the mediating and moderating mechanisms proposed in the conceptual model will elucidate how housing insecurity is connected to health and provide insight into preventive strategies to ameliorate the adverse effects of housing insecurity on biological health among aging adults.

## 1 Introduction

Housing insecurity (HI) is a pernicious chronic stressor and social determinant of health equity that threatens shelter and ontological security (1–3). HI reflects multiple dimensions, including housing stability (e.g., eviction), affordability (e.g., housing cost burden), quality (e.g., utility issues), safety (e.g., environmental toxins in the home), and homelessness (4). HI contributes to adverse mental and physical health (e.g., depression, chronic physical conditions) (5–12), with emerging evidence connecting HI to physiological and epigenetic indicators (e.g., C-reactive protein, epigenetic aging) (13–15).

While associations between housing and health are well established, causal pathways by which housing can contribute to health, particularly physiological and epigenetic indicators, are poorly understood (2, 16, 17). The scarcity of evidence on these pathways motivates this narrative review. This review borrows from and expands on the transdisciplinary stress process framework, adapting it to the HI-health linkage context (18).

Stress can be defined as a sum or cumulation of wear and tear on the body caused by vital reactions to stressors (19). Types of stressors include major life changes (e.g., divorce, job or housing loss) and quotidian stressors (which include chronic stressors or persistent/recurring life difficulties such as repeated stress around making rent/mortgage payments; as well as daily hassles) (19). The traditional stress process framework describes how disruptive life events (e.g., HI) can impact mental health through mechanisms including economic strain, self-esteem, self-efficacy, coping resources, and social resources (20). Across older and newer stress process models, there are three predominant domains: (1) sources of stress (i.e., stressors), (2) mediators/moderators of stress, and (3) manifestations of stress (18, 20–22). This review employs this existing framework to trace pathways linking HI (stressor) with physiological/epigenetic indicators of health (manifestations of stress), especially among aging adults (23, 24). We propose a conceptual model based on the synthesis of literature in stress, HI, and health fields, and position the literature within the context of our proposed conceptual model. In doing so, we also discuss fundamental concepts and key pathways in the linkage between HI and health markers, in ways that offer valuable insights for policymakers, researchers, and practitioners working to reduce health disparities and promote equitable health outcomes.

## 2 Methods

### 2.1 Conceptual framework

**Figure 1:**
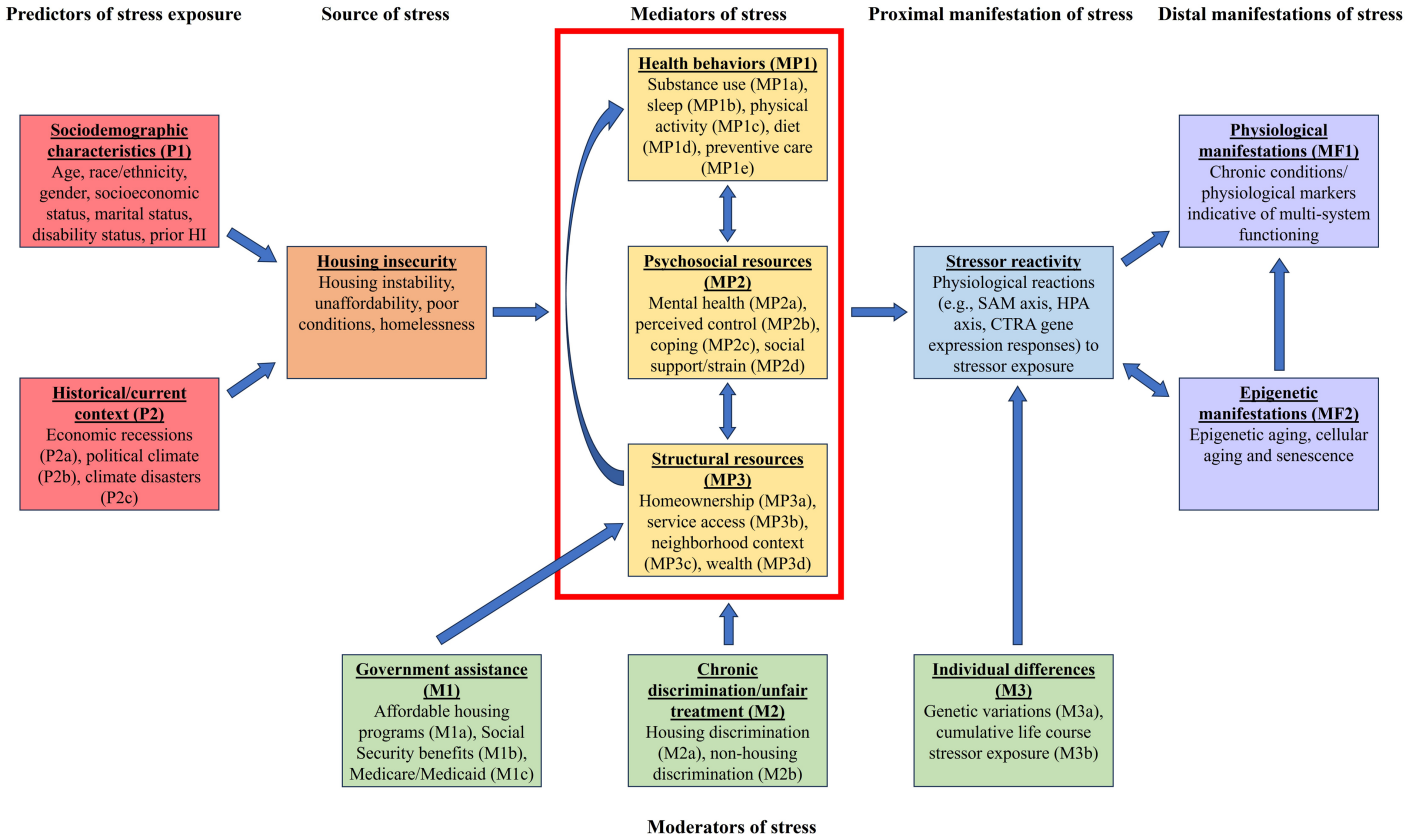
Conceptual model. Some arrows have been omitted from figure for clarity. For example, government assistance (M1) and chronic discrimination/unfair treatment (M2) may also modify exposure to housing insecurity. SAM, sympathetic-adrenal-medullary axis; HPA, hypothalamic–pituitary–adrenal axis; CTRA, Conserved Transcriptional Response to Adversity.

A modified conceptual model based on adapted stress process models (18, 21) and a body of literature examining the connections between HI and biological outcomes formed the basis for this narrative review. The model is shown in Figure 1. Moving from left to right, the red boxes to the left describe predictors of stressor exposure, including sociodemographic characteristics (P1) and historical/current context (P2); the orange box characterizes the source of stress of interest, HI; the yellow boxes framed within the red enclosure (top center of Figure 1) illustrate the main distinct mediating pathways (though they can also be potentially mutually complementing pathways) between HI and stressor reactivity (the latter shown as the blue box). Stressor reactivity, a proximal manifestation of stress, refers to how an individual responds to the HI stressor, including physiological reactions (19). The stress literature indicates that the health implications, or distal manifestations, of stress are rooted in exposure to stressors and stressor reactivity (19). Thus, stressor reactivity leads to the physiological/epigenetic manifestations of health (in purple boxes), or distal manifestations of stress, toward the right of Figure 1 (MF1 and MF2). The green boxes (bottom center of Figure 1) indicate moderators of the three distinct mediating pathways as well as stressor reactivity. The arrow pointing from government assistance (M1) to structural resources (MP3) represents a modifying effect, such that access to government assistance may ameliorate or exacerbate associations between HI and structural resources. Chronic discrimination/unfair treatment (M2) also acts as a moderator between HI and each of the three mediating pathways within the red enclosure. Finally, individual differences (M3) are indicated as a moderator affecting how the mediating pathway effects (separately and in totality) translate to stressor reactivity.

Given the broad and interdisciplinary nature of this review, a narrative review format provided flexibility to pull literature across academic fields for the purpose of developing a transdisciplinary conceptual model linking housing insecurity to physiological and epigenetic manifestations of stress. The literature selected for this review included foundational and recent studies spanning multiple fields, including public health, psychology, sociology, and aging studies, and was identified through scientific databases including Web of Science, PubMed, JSTOR, and Google Scholar. Selected literature included primarily peer-reviewed empirical studies, literature reviews (including a few book chapters), and research reports published in the English language between 1981 and 2024. Literature for this review was primarily based in the United States sociodemographic, economic, political, and geographic context; a minimal number of studies came from other Western, educated, industrialized, rich, and democratic (WEIRD) countries. Table 1 provides a list of selected key literature that informed the development of the mechanisms described in the conceptual model, including the stress process in the context of aging adults and each component of the model.

**Table 1:** Summary of topics and relevant literature.

Topic	Examples	Key selected literature
Defining HI	Cost, conditions, consistency, context	(1, 4)
The stress process	Traditional model of stress	(20)
	Expanded models of stress	(21, 22)
	Transdisciplinary, integrative models of stress	(18)
The stress process and aging adults	SAVI model	(19, 31, 32)
Predictors of HI	Sociodemographic characteristics (P1)	(5, 88, 89)
	Historical/current context (P2)	(7, 39, 97)
Mediators of stress	Health behaviors (MP1)	(33, 100, 120)
	Psychosocial resources (MP2)	(7, 125, 129)
	Structural resources (MP3)	(24, 90, 124)
Moderators of stress	Government assistance (M1)	(38, 154, 157)
	Chronic discrimination/unfair treatment (M2)	(146, 160, 162)
	Individual differences (M3)	(170, 172, 178)
Proximal manifestations of stress	Differential physiological stressor reactivity	(19, 30, 32)
	SAM axis, HPA axis	(45, 48)
	Epigenetic stress processes	(46, 51, 52)
Distal manifestations of stress	Physiological manifestations of stress (MF1)	(44, 58, 61)
	Epigenetic manifestations of stress (MF2)	(72, 73, 76)

SAVI, Strength and Vulnerability Integration Model; SAM, sympathetic-adrenal-medullary axis; HPA, hypothalamic–pituitary–adrenal axis.

#### 2.1.1 The stress process, HI, and aging adults

Midlife, broadly encompassing 40–64 years of age (25–27), is often characterized by the middle stages of parenting, career peaking or early retirement, and social and personal responsibilities (26, 27). Midlife adults are sometimes called a “sandwich generation” with multiple roles, such as caring for children and aging parents simultaneously. Thus, stressors midlife adults face (e.g., HI) may have implications for multiple generations within a family, particularly given the increased number of multigenerational households in the United States (U.S.) (26, 28, 29). Midlife is also characterized by declines in physical/functional health and increased chronic conditions; simultaneously with growth in knowledge, experience, and emotional regulation (27). According to the Strength and Vulnerability Integration (SAVI) model, this pattern continues into older adulthood (age 65+), as less flexible physiological systems make older adults less adept at physiologically adapting to unavoidable stressors such as HI (19, 30–32).

While the number of life events declines with age, aging adults are experiencing more economic strain (including HI) than prior generations, partly because of circumstances such as the 2007–2009 Great Recession (GR) and COVID-19 pandemic (30, 32–38). Among midlife U.S. adults over 50, homelessness has increased by 11% in the past 10 years, and 1.3 million adults in this age group are estimated to be behind in housing payments (28, 29). Among adults 65 or older, over 30% are classified as low-income; and 26% of older adult homeowners and 54% of renters are cost burdened (spending 30% or more of their income on housing) (28, 34). In fact, the rates of cost burdened homeowners and renters in the 65+ age group are second only to the cost burden among those under 25 years old (34). The percentage of older adults who rent as opposed to owning a home has also increased to 22%, and many of these lifelong renters are forced to delay retirement due to vulnerability to rising rent prices and few legal protections as renters, which contributes to an increasing gap between aging renters versus homeowners (28, 34). Older renters also experience more mobility, which can indicate HI, compared to older homeowners (28). Homeowners who are 75 or older currently have the highest foreclosure rates compared to any age group in the U.S. (34), and additionally, among older adults (65+), homelessness is projected to triple across the next decade (35). Additionally, as COVID-19 pandemic-related eviction moratoriums and housing relief funding provided at a federal and local government level in the U.S. have declined since the peak of the pandemic, eviction rates have increased among vulnerable groups (37–40). Given the confluence of the increasing aging population in countries such as the U.S. (41) with increased vulnerability to HI, it is critical to understand the mechanisms linking these stressors to health outcomes, given that HI may increase stress and negatively affect the biological health of already vulnerable aging adults (25).

## 3 Results

We begin by discussing how stress becomes physiologically or epigenetically embedded, subsequently manifesting as indicators (markers) of health. Thus, we start from the proximal and distal manifestations of stress on the right side of Figure 1.

### 3.1 Stressor reactivity and indicators measuring distal manifestations of stress

Stress becomes physiologically and epigenetically embedded and manifests in multiple health markers (42–44). Two major physiological systems implicated in the *physiological embedding* of stress (MF1 in Figure 1) include the sympathetic-adrenal-medullary (SAM) axis and the hypothalamic–pituitary–adrenal (HPA) axis. In response to a perceived stressor, sympathetic nervous system (SNS) activation contributes to the “fight or flight” response, activating the adrenal medulla and sympathetic neurons to secrete epinephrine and norepinephrine in the bloodstream, which increases heart rate and blood pressure, dilates pupils, and prepares the body to deal with acute threats (19, 43, 45, 46). This process of SAM axis activation is evolutionarily beneficial in the context of acute stressors, but perpetual activation can cause harm through chronically elevated systolic blood pressure and exacerbating health conditions (19, 47).

The HPA axis is also activated by stressors but is characterized by the release of corticotrophin-releasing hormone (CRH) from the hypothalamus, which contributes to the release of adrenocorticotropin hormone (ACTH) and arginine vasopressin (AVP) from anterior and posterior pituitary glands, respectively (19, 48). ACTH causes the release of glucocorticoids from the adrenal cortex, which restricts the continued release of CRH and ACTH, limiting further glucocorticoid production; while AVP supports the “flight or fight” response in situations of stress or threat (19, 49). While the HPA axis contributes to short-term physiological adaptation, chronic activation may contribute to chronic illness, restricted immune response, and damage to hippocampal neurons (19). With age, adults become more vulnerable to inflated HPA axis response to stressors (19).

Another potential and complementary mechanism of stressor reactivity is gene expression. Individual differences in the expression levels of genes contribute to the differential encoding of proteins which mediate immune-related responses, such as inflammatory cytokines or antimicrobial molecules (50). A specific example of gene expression stressor reactivity patterns is the conserved transcriptional response to adversity (CTRA), which describes a physiological pattern characterized by the upregulation of genes related to inflammation and the downregulation of genes implicated in interferon and antibody responses (51, 52). The CTRA composite score is characterized by pro-inflammatory gene expression levels across up to 19 genes (which form the CTRA inflammatory subcomponent score) minus antiviral gene expression levels across up to 34 antiviral genes (which form the CTRA antiviral subcomponent score) (50, 53). CTRA is activated by stressors and adversity across the life course (51, 53). Expression of CTRA inflammatory genes and decreased expression of antiviral genes may contribute to physiological inflammation and susceptibility to chronic conditions and viral infections (54, 55).

Several indicators of physiological dysregulation are commonly assessed. Chronic physical conditions are a common indicator of physiological embedding of stress, as they have been shown to be associated with stress-related physiological deterioration through mechanisms such as inflammatory pathways (56, 57). Cortisol is a key stress biomarker associated with the negative feedback loop of the HPA axis; it limits the production of CRH and ACTH and impacts behavior, cognition, immunity, and metabolism, thus affecting various chronic physical health conditions (58). Cytokines are biomarkers associated with the immune system, including pro-inflammatory cytokines interleukin (IL)-6, IL-1β, and tumor necrosis factor (TNF)-*α*; along with C-reactive protein (CRP), a plasma protein produced in the liver (59), these markers are associated with chronic inflammation associated with disease, and are reactants to chronic and acute stress (44, 58). A more comprehensive measure of physiological strain utilized in stress research is allostatic load (AL). AL occurs when “status quo” allostatic processes (active regulatory processes that adapt to physiological needs and environmental stimuli to maintain an organism’s stable internal environment) (47, 60) wear out or do not appropriately turn off, leading to the maladaptation of physiological functions. AL comprises multiple indicators (between 10 and 24, ranging from blood pressure, waist-hip ratio, heart rate, cortisol, epinephrine, inflammatory markers, etc.) across multiple physiological systems (including the cardiovascular, sympathetic nervous, parasympathetic nervous, HPA, inflammatory, lipid metabolism, and glucose metabolism systems) that are aggregated to create a composite score of physiological functioning (47, 61–64).

Stress can also become *epigenetically embedded* (MF2 in Figure 1) through similar mechanisms as the physiological markers previously discussed. HPA axis activation and resulting increased cortisol levels can potentially induce DNA double strand breaks (DBSs), leading to activation of DNA repair proteins in certain cells (65, 66), cellular senescence or epigenetic changes, and accelerated epigenetic aging (46, 67, 68). Stress can contribute to DNA modifications, including DNA methylation (DNAm), which occurs when a methyl group is added to, typically, a cytosine followed by a guanine base (CpG site), although DNAm can also occur at cytosines followed by non-guanine bases; or even at bases that are not cytosine at all. When DNAm occurs, particularly at CpG sites, gene expression can be impacted by being dampened, whereas DNA demethylation is generally associated with amplification of gene expression; indicating a potential bidirectional relationship between DNAm and stressor activity pathways of gene expression, including CTRA gene expression (42, 69–72). Methylated cytosines are also especially vulnerable to mutations, which may even contribute to genomic changes (72). DNAm patterns have led to the development of epigenetic “clocks” estimating biological age based on DNAm at sites associated with markers of aging; accelerated epigenetic aging refers to when the estimated biological age is older than chronological age, and is predictive of morbidity and mortality risk (73–80). Other epigenetic mechanisms include chromatin modifications and histone protein modifications, which may increase or decrease access to DNA, and lead to nucleosome modification through incorporation of histone variants and added post-translation modifications; noncoding RNAs may regulate transcription; and RNA modifications occur when chemical groups are added to RNA nucleotides (72). Additionally, another important epigenetic manifestation of stress in aging is cellular aging and senescence, which is affected by DNAm, chromatin and histone modifications, and DNA damage signaling; these mechanisms mediate the induction and maintenance of cellular aging and senescence through their role in regulating genome architecture and gene expression (81–83). Cellular aging and senescence are associated with aging-related pathologies, including cancer and cardiovascular disease; thus, an arrow from epigenetic manifestations (MF2) to physiological manifestations (MF1) is illustrated in Figure 1 (46, 83–85). However, there is currently limited empirical work outlining connections between social and environmental stressors contributing to oxidative stress and cellular aging and senescence (82, 86, 87). Thus, among these epigenetic manifestations, this review will primarily focus on DNAm given its role in biomarkers indicative of epigenetic aging, as well as the relatively extensive current empirical evidence connecting social and environmental stressors to DNAm and its manifestation of epigenetic aging.

### 3.2 Predictors of HI

We now proceed from left to right in Figure 1, mapping our discussion to the conceptual model.

*Sociodemographic characteristics* (P1 in Figure 1) can predict HI exposure. Older adults have lower HI than midlife adults, perhaps partly because family support networks are more willing to provide housing or financial assistance for older adults and increased government support (5, 88). Minoritized racial/ethnic groups are much more vulnerable to experiencing HI due to historic redlining, housing discrimination, and gentrification than non-Hispanic whites (88–92). Being female, unmarried, having lower socioeconomic status (e.g., education, income), having a disability or impaired mobility which may contribute to difficulty transitioning out of physically inaccessible housing (particularly for older adults), and prior HI are other sociodemographic predictors of HI (5, 88, 89, 91, 93, 94).

The second predictive category for HI exposure is historical or current economic, political, or climatic contexts (P2 in Figure 1). HI surges in the aftermath of *economic recessions* (P2a in Figure 1), such as the GR (7, 29, 95, 96) or COVID-19 pandemic (37, 39). These economic contexts have contributed to the U.S. housing sector experiencing unprecedented unaffordability (1). The confluence of these conditions has contributed to increasing missed rent and home payments, informal evictions, formal evictions, and homelessness rates (7, 29, 95). Additionally, contexts of *political climate/violence* (P2b in Figure 1) or *climate disasters* (P2c in Figure 1) can contribute to damaged or destroyed housing or the need to leave housing behind (e.g., displaced refugees) (97–99).

### 3.3 Mediating pathways linking HI and physiological and epigenetic health manifestations

Stress process models describe how primary stressors (e.g., HI) can proliferate and contribute to secondary stressors, which are denoted as “Mediators of stress” in Figure 1 (21). Competing and complementary pathways may act as potential mediators in the HI-health relationship. Three particular mediating pathways that stand out in the literature include those associated with (1) health behaviors, (2) psychosocial resources, and (3) structural resources. These are discussed in turn in the subsequent sections.

#### 3.3.1 Mediating role of health behaviors (MP1)

Stress related to economic strain, such as HI, can contribute to risky health behaviors. According to the despair hypothesis (8, 100, 101), social disparities in longevity have increased due to the vulnerability of the less educated to labor market changes, which contribute to material hardship such as HI and lesser job and economic mobility prospects, increased distress, and thus adverse health behaviors, specifically *substance use* (MP1a in Figure 1) (100). Chronic substance use can persistently activate corticotropin-releasing factor (CRF; implicated in the HPA axis), which enhances stressor reactivity and can eventually result in increased AL (102). Additionally, chronic substance use among older adults has been indicated to be associated with poorer mental and physical health (103); and across studies has been indicated to be a potential mechanism linking homelessness among older adults to poorer mental and physical health (104).

Housing instability contributes to *insufficient sleep* (MP1b in Figure 1) through increased exposure to the elements, noise, danger, and absence of privacy (33, 101, 105–110). In addition to physical obstacles to sleep, housing instability and material hardship contribute to psychological distress, which exacerbates poor sleep (101). Insufficient sleep increases the risk of mental health disorders, chronic physical conditions (e.g., cardiometabolic diseases, cancer, cognitive issues, dementia), and mortality (101, 111–115). Given that sleep is predictive of chronic conditions and is associated with physiological inflammation and accelerated epigenetic aging, sleep may be a critical health behavior linking HI with physiological and epigenetic markers of well-being among aging adults (116, 117).

HI may increase *physical inactivity* levels (MP1c in Figure 1) because of lower access to safe areas and greenspace to exercise and be physically active, and psychological strain from HI and general economic insecurity causing “tradeoffs” of time and resources from healthy behaviors to more immediate “survival” or “coping” behaviors (11, 118). Physical activity is associated with improved psychological and physical/physiological health (self-rated and biomarker indicators) among older adults (119, 120), which makes it a necessary health behavior to consider in the HI-health relationship.

HI often co-exists with *food insecurity* (MP1d in Figure 1), given that those who are struggling with housing affordability or live in food deserts have lower access to quality affordable foods (101, 121, 122). Lack of adequate nutrition or reliance on unhealthy fast foods (disproportionately an issue for lower socioeconomic status populations) can contribute to malnutrition, chronic conditions, and health disparities (121). Aging adults living in lower-income neighborhoods (who may likely be experiencing HI) have higher AL due to chronic stress and cumulative damage to physiological regulatory systems, which may be partially accounted for by fast food consumption, as well as smoking and exercise habits (24).

The evidence above indicates that health behaviors may partially contribute to the relationship between HI and health outcomes. In addition to the more commonly discussed health behaviors of substance use, sleep, physical activity, and eating, housing-insecure adults are six times more likely to delay *preventive medical care* (such as doctor visits; MP1e in Figure 1) due to cost than non-housing insecure adults and have difficulty managing intensive chronic conditions (e.g., diabetes) because of difficulty preparing adequate food, maintaining medication routines, or keeping medications stored appropriately and safely versus those who are housing secure (8, 123, 124).

#### 3.3.2 Mediating role of psychosocial resources (MP2)

Studies show that economic and HI issues contribute to poorer psychological wellbeing, including increased rates of anxiety, depression, and suicidal ideation. Recession events such as HI can also contribute to higher rates of relationship conflict and poorer relational functioning (125). Such psychosocial stress associated with HI can mediate the relationship between HI and physiological/epigenetic manifestations of health. HI experiences (e.g., eviction, foreclosure) contribute to poorer self-reported mental health, anxiety, depression, and increased suicide rates (7, 36, 126–129). *Mental health* (MP2a in Figure 1) and associated affective disorders are associated with AL through stress processes (130). HI may also be associated with health by affecting *perceived control* (sometimes referred to as mastery; MP2b in Figure 1) (6, 9, 20, 21). The reserve capacity model discusses how psychosocial resources (e.g., perceived control) are associated with better health outcomes and may buffer effects of social disadvantage, such as HI and economic strain (131); empirical evidence shows perceived control is a buffer between economic hardships and psychological and physical health (131, 132); and may be among eudaimonic wellbeing factors associated with CTRA down-regulation (53, 133). Another mediator between HI and biological health is *coping* (behavioral or cognitive response to a stressor that may ameliorate harm caused by the stressor; MP2c in Figure 1) (20, 21). HI may lead to reductions in time, emotional, and financial resources to engage in coping (19). Coping strategies such as mindfulness techniques can affect emotional regulation for aging adults and dampen physiological stress responses (19).

*Social support or strain* (MP2d in Figure 1) is another important psychosocial resource beyond mental health and individual emotional regulation strategies. Economic events such as HI can contribute to higher rates of relational conflict and poorer functioning (125). Ascigil et al. (125) describe the vulnerability stress adaptation model in the context of marital relationships, such that stressors outside marriage (e.g., adverse recession exposures or HI) contribute to poorer couple relationships due to lower quality communication and increased disagreements and tension, which then contribute to poorer mental health (e.g., negative affect, affective disorders). Given that midlife is often characterized by social roles and relationships, and that midlife adults often provide intergenerational support, exposure to HI can be particularly stressful during this life stage as it may influence relationships with emerging adult children and aging parents (26). Social support is protective against adverse health outcomes, whereas social strain or isolation is associated with an increased likelihood of CTRA activation, chronic conditions, accelerated epigenetic aging, and mortality (53, 134–136). Studies have indicated that, among older adults experiencing homelessness, loneliness is a potential mechanism that contributes to functional physical decline (104).

As discussed in the previous section, psychosocial distress may contribute to adverse health behaviors, including substance use, poor sleep, and changes in physical and dietary patterns. These negative health behaviors may influence psychosocial resources, such as mental well-being and interpersonal relationships (8, 100, 101). Thus, in the conceptual model, a bidirectional arrow is indicated between health behaviors and psychosocial resources.

#### 3.3.3 Mediating role of structural resources (MP3)

High housing costs, a significant contributor to HI, contribute to health risks through various mechanistic pathways, including material deprivation, forcing tradeoffs between housing and other goods or services for living, and leading to potentially forgoing housing and neighborhood quality to secure financially manageable housing (118, 137). Unsurprisingly, subsidized housing is associated with better health due to limiting HI and some potential tradeoffs of resources (e.g., money to afford a car or transportation, and educational opportunities, etc.) (1, 11, 137). Studies also show that unaffordable housing is associated with increased odds of chronic conditions (e.g., hypertension, arthritis), and this association is more robust for renters than homeowners (11); additionally, renting rather than homeownership is associated with higher CRP and accelerated epigenetic aging (14, 15). HI is more prevalent among renters than homeowners (29). Evidence indicates structural assets such as *homeownership* (MP3a in Figure 1) protect against health deterioration among those experiencing HI.

Unstable housing is associated with *less service access* (MP3b in Figure 1), including access to healthcare and insurance (1), which may affect the use of mental and physical preventive care (1, 37, 138). Affordable and reliable public transit access may also mediate the HI-health relationship by affecting access to resources such as parks or gyms to maintain physical exercise, healthy food (e.g., supermarket availability), healthcare, and travel to places of employment (1).

*Neighborhood context* (MP3c in Figure 1) is another potential structural resource mediator between HI and health: access to greenspace increases walkability and physical activity, which can reduce the risk of obesity, as well as lower general stress levels, healthier cortisol levels, and potentially epigenetic changes associated with better health (1, 139, 140); exposure to environmental toxins (e.g., proximity to pollution sites), and other subjective and objective aspects of neighborhoods (e.g., safety, poverty) can contribute to chronic physical conditions (e.g., asthma, neurocognitive impairment, cancer, mortality), AL, and accelerated epigenetic aging (14, 24, 141–143). These aspects of neighborhood contexts are also associated with mental health (144). Because low-income and housing insecure groups are especially vulnerable to inadequate neighborhood conditions and environmental hazards, these contextual characteristics may be an essential mechanism in the relationship between HI and biological health markers (18, 145).

Finally, material resources, such as access, or lack thereof, to intergenerational *wealth* (MP3d in Figure 1) may mediate the relationship between HI and physiological health. HI and loss of financial resources through renting can contribute to wealth loss (90, 92, 146–148). However, if housing insecure individuals or families can tap into such resources and sell assets or receive financial support from the family, this may lessen the severity of HI and its health implications (90, 149–151). Therefore, psychosocial resources, such as the quality of familial relationships, may predict the ability to access familial wealth, providing further evidence for the intrinsic interconnectedness of these mediating mechanisms.

### 3.4 Moderating factors between HI and physiological and epigenetic health manifestations

In addition to the three mediating pathways just discussed, there are three moderating factors, as discussed below, that influence the relationship between HI and physiological/epigenetic health manifestations: (1) government assistance, (2) chronic discrimination and unfair treatment, and (3) individual characteristics.

#### 3.4.1 Moderating role of government assistance (M1)

Access to government assistance may ameliorate the relationship between HI and biological health outcomes. Older adults at retirement age are eligible to receive and access *affordable housing programs* (M1a in Figure 1) and government benefits (e.g., *Social Security benefits* and *Medicare/Medicaid*; M1b and M1c in Figure 1) (26, 152, 153). Across age groups, access to rental assistance programs reduces odds of poor self-reported mental and physical health, and risk of uncontrolled diabetes and high hemoglobin A_1c_ (154–157); and subsidized housing is associated with better health due to limiting HI and some potential tradeoffs of resources (e.g., money to afford a car or transportation, educational opportunities, improved access to healthcare/insurance) (11). Additionally, eviction moratoriums imposed during the COVID-19 pandemic were shown to reduce HI and even limit viral spread (37, 38). Access to other government benefits including unemployment benefits, Social Security, and health insurance, can decrease HI through financial resources, and limit health issues through access to preventive care (152, 158, 159). Through pathways of improvement in structural resources, which can then confer benefits to health behaviors and psychological wellbeing, government assistance may reduce the effects of HI on biological health.

#### 3.4.2 Moderating role of chronic discrimination and unfair treatment (M2)

Chronic exposure to discrimination or unfair treatment across *housing-related* (M2a in Figure 1) (146) and *non-housing* (M2b in Figure 1) domains is associated with increased AL (160), inflammatory gene expression (161), and accelerated epigenetic aging (162), especially among minoritized or marginalized adults who are disproportionately exposed to oppression and experiencing double jeopardy of socioeconomic status deprivation alongside discrimination according to the minority poverty hypothesis (1, 163–165). The history of housing-related discrimination in the U.S. may particularly affect older adults who were alive during the urban renewal programs of the 1950s which displaced close to 1 million racial and ethnic minorities (1, 166); as well as exposure to extreme housing discrimination practices of loan discrimination and redlining, which resulted in non-white racial and ethnic groups being blocked from residency in certain neighborhoods (1, 92). While redlining practices were technically outlawed as part of the Fair Housing Act of 1968, in practice, redlining and other discriminatory housing practices still occur across the country (92). However, those older adults who lived through these periods of extreme housing discrimination prior to the Fair Housing Act of 1968 and other subsequent fair housing laws passed in later decades may have a unique historical exposure to housing-related discrimination across the life course; and these differential exposures across the current generation of older adults in the U.S. make studying the relationship between housing discrimination and health outcomes in this age group particularly salient.

Exposure to discrimination may modify the relationship between HI and the three mediating mechanisms of interest, such that discrimination in the face of HI may exacerbate adverse health behaviors (167), reduce psychosocial resources such as mental wellbeing (168), and reduce access to structural resources such as homeownership or living in safe and higher-income neighborhoods (146); thus leading to biological health disparities.

#### 3.4.3 Moderating role of individual characteristics (M3)

Individual characteristics, including *genetic considerations* (M3a in Figure 1) and *cumulative life course stressor exposure* (M3b in Figure 1), may act as modifiers (by influencing stressor reactivity) among the pathways linking HI with biological outcomes. Personality in adulthood can form as a direct manifestation of early life temperament (developmental stability or developmental elaboration), or later experiences or role changes can layer new dimensions onto an individual’s genetically influenced temperament to affect personality (developmental change or the social investment hypothesis) (169–171). Gottlieb’s (172, 173) developmental psychobiological systems framework conceptualizes bidirectional interactions between genotype, neural activity, behavior, and environment on individual development. Extant work has indicated associations between more neurotic personality types and increased threat appraisal and stressor reactivity when faced with a stressor compared to other Big Five personality types (174–176). Additionally, multiple stressor exposures can lead to a cumulative, compounding, and exponential impact on stressor reactivity and biological weathering, compared to an additive effect (1, 177, 178). Thus, the combination of life experiences (e.g., how one has previously responded to stressors) and genetic variations may influence aspects of personality and how individuals differentially react to stressors (19, 179).

Given that those who experienced HI and neighborhood/economic disadvantage during childhood and adolescence are more prone to experiencing HI and economic strain later in life (93, 177, 178, 180–182), it is critical to take a life course human development perspective when examining HI and health. Housing and neighborhood environment and economic hardship earlier in the life course have also been shown to have crucial implications for later life health outcomes, including mental health, physiological health markers, epigenetic aging, and mortality risk (10, 183–189). Because the effects of earlier HI and potential repeated exposures may cumulate across time to influence health risk in later adulthood, understanding these trajectories from a life course perspective can highlight how multiple housing-related stressor exposures across time may lead to a compounding effect on stressor reactivity and biological weathering for housing insecure older adults (1, 177, 178).

## 4 Discussion

HI meets all the key criteria for a stressor. It disrupts homeostasis by threatening a basic need, is perceived as a significant threat by those affected, and demands continuous adaptation efforts, leading to adverse mental, physical, and biological health outcomes. This review is significant for several reasons:

First, it introduces multiple dimensions of HI, including housing stability, affordability, quality, and safety, thus offering a holistic understanding of how the different aspects of HI contribute to stress and impact health as a social inequality.

Second, it builds upon existing stress process models to elucidate the pathways through which HI affects health: through risky health behaviors, psychosocial stress, and structural strain. Additionally, it illustrates how HI may activate stress response systems (e.g., SAM and HPA axes). Understanding these pathways is crucial for developing targeted interventions to mitigate HI’s health impacts.

Third, it highlights the vulnerability of certain populations, such as marginalized and disadvantaged aging adults, to the long-term health impacts of HI (e.g., chronic illnesses, epigenetic changes), underscoring the need for tailored strategies to address the challenges faced by these populations to promote health equity.

Fourth, by synthesizing existing research, it provides evidence-based insights that can inform policymakers and stakeholders and guide the development of policies and programs aimed at reducing HI and its health disparities to promote public health among aging adults, such as increasing housing assistance or initiatives to help older adults age in place (190–193). Such interventions may also have significant economic implications. Projections indicate that housing instability among families with children under 18 years of age (which may include some midlife adults) will result in a $111 billion increase in healthcare and education costs in the U.S. over the next decade (194). Given the rise in HI and healthcare costs associated with chronic conditions among aging adults, there will be substantial economic costs associated with HI over the next several decades in the U.S. as the proportion of midlife and older adults continues to increase in the population (34, 190).

## 5 Conclusion

This review provides a critical and detailed evidence-based analysis of the complex relationship between HI and health. Incorporating insights from public health, psychology, sociology, and other fields, it provides an interdisciplinary perspective that enhances the robustness of the analysis and supports comprehensive strategies to tackle HI and its health impacts. Identifying key pathways and highlighting the experiences of vulnerable populations offer valuable insights for policymakers, researchers, and practitioners working to reduce health disparities and promote equitable health outcomes. Future directions of this work should include empirically testing the proposed mediating/moderating mechanisms in the conceptual model to elucidate mechanisms by which HI is associated with health outcomes among aging adults.
